# Spreading of COVID-19: Density matters

**DOI:** 10.1371/journal.pone.0242398

**Published:** 2020-12-23

**Authors:** David W. S. Wong, Yun Li

**Affiliations:** 1 Department of Geography and GeoInformation Science, George Mason University, Fairfax, VA, United States of America; 2 NSF Spatiotemporal Innovation Center, George Mason University, Fairfax, VA, United States of America; Institute for Advanced Sustainability Studies, GERMANY

## Abstract

Physical distancing has been argued as one of the effective means to combat the spread of COVID-19 before a vaccine or therapeutic drug becomes available. How far people can be spatially separated is partly behavioral but partly constrained by population density. Most models developed to predict the spread of COVID-19 in the U.S. do not include population density explicitly. This study shows that population density is an effective predictor of cumulative infection cases in the U.S. at the county level. Daily cumulative cases by counties are converted into 7-day moving averages. Treating the weekly averages as the dependent variable and the county population density levels as the explanatory variable, both in logarithmic scale, this study assesses how population density has shaped the distributions of infection cases across the U.S. from early March to late May, 2020. Additional variables reflecting the percentages of African Americans, Hispanic-Latina, and older adults in logarithmic scale are also included. Spatial regression models with a spatial error specification are also used to account for the spatial spillover effect. Population density alone accounts for 57% of the variation (R-squared) in the aspatial models and up to 76% in the spatial models. Adding the three population subgroup percentage variables raised the R-squared of the aspatial models to 72% and the spatial model to 84%. The influences of the three population subgroups were substantial, but changed over time, while the contributions of population density have been quite stable after the first several weeks, ascertaining the importance of population density in shaping the spread of infection in individual counties, and in their neighboring counties. Thus, population density and sizes of vulnerable population subgroups should be explicitly included in transmission models that predict the impacts of COVID-19, particularly at the sub-county level.

## Introduction

In the midst of the COVID-19 pandemic, we are still uncertain about the pathways how one may contract the virus [1–3]. Despite the lack of this critical knowledge, numerous studies, published and on-going, try to develop models to better predict various disease statistics, such as the reproduction number (R_t_), but ultimately to predict the size of the infected population and causalities. With no certainty on the details of the transmission processes, social distancing, which is a misnomer and should be replaced by physical distancing or separation [4, 5], has been regarded as one of the effective means to combat the spread of virus [6]. Increasing evidence suggested that not just droplets, but aerosolization of viral particles is a possible transmission mechanism [7, 8]. Thus, distancing is not a foolproof to avoid contracting the virus, although it is still strongly recommended. Different specific distancing guidelines are required for different localities to accommodate the local geographical contexts. For instance, the 2-meter separation adopted in the U.S. is impractical in some Asian cities such as Tokyo and Hong Kong due to the high overall population density levels. Be able to maintain distancing outside of one’s household is dependent upon a variety of factors, including living environments (apartments versus single-family dwellings), settings of physical infrastructures (public transportation in inner cities versus automobiles in suburbs), and work arrangements (IT consultants working at home versus employees in food service industry). Nevertheless, the underlying principle is to avoid close contact.

Being able to keep a certain distance apart from each other is not a corollary of avoiding contact, as making contacts is partly a behavioral issue and partly an objective environmental issue constrained by population density. Conceptually, relationships between population density and contact rates are nonlinear and operate differently at different geographical scales [9]. Empirical studies using the 1918 influenza and pneumonia mortality data of the U.S. at the state level and data of cities and towns in England and Wales do not offer very strong correlations between total mortality and population density levels [9]. However, a review of large-scale spatial models of disease transmission concludes that models including population density are more parsimonious [10].

The U.S. Center for Disease Control and Prevention (CDC) has been referring to a dozen or so models to inform the public about the trajectories of the pandemic and to support decision-making [11]. These models offer a variety of estimates, including daily new cases and deaths, and the cumulative statistics. Some of these models (e.g., the ones by Georgia Tech and University of Texas-Austin) rely on sophisticated computational and data mining methods, using a data-driven approach to learn about the association between mobility and mortality to predict the number of deaths. Other models focus on estimating the transmission rates (e.g., the ensemble of four models developed by the Imperial College, London, the models developed by Columbia University and the University of Chicago). Some models include more empirical population and geographical variables (e.g., the Notre Dame’s agent-based model and the Northeastern University’s GLEAM model.) While some of these models consider people’s mobility, they consider only the frequencies and/or distances of travels, but fail to account for the potential contacts that the travelers might have made, which are largely a function of population density in the region. Although at least two models (the IHME and MIT models) consider population density, they use state-level density levels, ignoring the significant intra-state variation of density.

Despite the fact that numerous studies acknowledge the importance of population density in modeling pathogen transmission through its controls of contact rates or interaction (please refer to the volumes of studies reviewed in [9, 10]), few studies employ population density directly. The study by Hu et al [9] is an exception, while most studies are similar to the one by Tarwater and Martin [12] in which another variable, in this case, the average number of contacts with susceptible individuals per infectious individual, was used as a proxy of population density.

Population density has long been studied in urban economics and geography, typically at the intra-urban scale [13, 14]. Studies have modeled the spatial structure of population density in urban systems, historically shifting from a monocentric to polycentric structure [15–17]. The importance of population density in controlling population growth has been demonstrated at both the state and county levels in the U.S. [18]. There is a need to ascertain the importance of population density in modeling the spread of diseases. The main objective of the current study is to evaluate the influence of population density on COVID-19 infection in the U.S. More specifically, the objective is not to estimate the transmission rate, but to assess the role of population density on the total impacts of the infection–i.e., the totals of infected populations. One may postulate that counties with larger populations are likely to have more cases, and these counties with more people also have higher population density levels. Based on the 2010 Census data of the 3,144 U.S. counties excluding Puerto Rico, the correlation between population size and population density is only 0.33, a moderately low level. Thus, a connection between population density and total infected cases has yet to be established at the county level.

While the pandemic may be understood from multiple perspectives, one may perceive the pandemic as a spatiotemporal process operated at multiple geographical scales with humans as the vectors spreading the disease. The infection intruded the U.S. starting from a few entry points, which may be regarded as seeds. These entry points, assuming that the disease was imported, are major international transportation hubs or cities. As the infection spread from these seeds to smaller settlements, more cases continued to import through major population centers. It is not the intent of this paper to address the detailed spatiotemporal diffusion of the virus. Our study intends to assess the influence of population density in controlling the spread of COVID-19 infection at the macro-geographical scale over time. Thus, we adopt a spatiotemporal framework, evaluating the importance of population density in affecting the infection level over time, from the initial importation period to the time passing the peak of the infection “curve.”

Based on the U.S. situation, we found that population density levels at the county level can increasingly explain the variation of cumulative cases across counties as the epidemic progressed. In addition, we found significant spatial spillover effect of infection at the county level. Accounting the spatial spillover, over 76% of the variation of cumulative cases among counties can be explained by population density alone around week 13 of the pandemic. While adding sizes of older adults, African American and Hispanic populations in logarithmic scale to the models raised the explanatory power to about 84%, population density still play a significant and steady role in controlling the size of infected population.

## Materials and methods

### Data

Many studies of COVID-19 published recently and many models referred to by CDC used the data provided by the Johns Hopkins University Coronavirus Resource Center (https://coronavirus.jhu.edu/). We acquired that dataset and examined the confirmed cases by counties in the U.S. from February to May, 2020 (https://github.com/CSSEGISandData/COVID-19). We found that for certain weeks, the data have gross errors. For this study, we used the data provided by USAFacts instead (https://usafacts.org/). Data from this source have been used by several portals, such as the spatiotemporal rapid response gateway to COVID-19 (https://covid-19.stcenter.net/), US COVID Atlas (https://geodacenter.github.io/covid/) provided by the University of Chicago’s Center for Spatial Data Science (CSDS) and Databricks COVID-19 resource hub(https://databricks.com/databricks-covid-19-resource-hub). Because the current study hypothesizes that population density is a major factor influencing the distribution of infection across counties, the outcome variable used, different from some studies that predict new cases daily or over a period, is (the logarithm of) the number of cumulative confirmed cases by counties. This indicator reflects reasonably the magnitude of morbidity impact of the epidemic. In addition, many studies and models use the total numbers of cases [11, 12], as these numbers can inform the public about the severity of the outbreak.

As our objective is to assess the influence of population density as the epidemic progresses in the U.S., ideally the study should use the population counts in 2020 to derive population density. Although the U.S. Census Bureau conducted the decennial census in April 2020, the data are not available yet. Therefore, we use the county population counts of 2010 decennial census to compute the population density of each county. Despite we do not have the most updated population counts, the variation of density across enumeration units is more important than the actual counts to assess the influence of population density on the numbers of confirmed cases. In addition, our units of analysis are counties. Population counts at the county levels between 2010 and 2020 should be different, but the relative differences should not have changed dramatically over a decade. One may recommend using the American Community Survey (ACS) data to derive population density. However, the latest release of ACS provides only 1-year estimates for 2018, not 2020. Also, errors in ACS estimates are not uniform across areal units, and thus, errors of estimate need to be considered when using these ACS estimates [19]. This requirement would complicate the use of ACS estimates tremendously in our study. Therefore, ACS data were not used.

Recent reports indicate that several population subgroups face disproportional burdens of contracting COVID-19 These groups are older adults, Latina-Hispanics, and African Americans. These subgroups are likely more economically disadvantaged. The two racial-ethnic minority groups in general have larger household sizes due to the multi-generational structure, raising the vulnerability of infection. Covaried with their occupation characteristics, these two subgroups have higher exposure risks than other population subgroups. Again, we obtained the population counts of these subgroups from the 2010 decennial census data at the county level, and the computed the percentages of older adults (Old), Latina-Hispanics (Hisp), and African Americans (AA).

## Methods

Although we have cumulative counts of confirmed cases by day starting from late January, these frequencies have significant day to day fluctuations. To remove the fluctuation and derive a more stable trend, many authorities have been reporting 5-day or 7-day moving averages. In our analysis, we used the 7-day moving average, which potentially accounts for the incubation period and the lapse between the time when symptoms first appear and the infection being diagnosed [20]. To ensure that the frequency distribution will not be influenced severely by outliers, we took a natural logarithm transformation of daily confirmed cases [20]. Specifically, we took the natural logarithm of (x_i_ + 1), where x_i_ is the cumulative count of confirmed cases on date *i*. Adding 1 to the frequency is to avoid taking the natural logarithm of 0 (which is undefined) at the expense of inflating all counts by 1. These natural logarithms of cumulative counts for seven consecutive days were used to compute the 7-day moving average. Therefore, this indicator is labeled as ma(lct). As the 7-day moving averages remove day-to-day fluctuation, there is no need to analyze the changes in frequencies from day to day. Thus, we used the ma(lct) value of every seven days to cover the study period from January 22 (week 1 in our data) to May 20 (week 18), 2020. However, before March, fewer than thirty counties have confirmed cases. Therefore, some of the statistical analyses used only data starting from March 4, 2020 (week 7).

Treating ma(lct) as the dependent variable, which varies over the 18 weeks, the natural logarithm of population density is the main independent variable. Population density is taken in the logarithmic scale because of its skewed distribution across U.S. counties. These two variables formed a simple regression model, which was estimated weekly. The other population variables in percentages, aligned with the logarithmic scale of the population density and cumulative count variables, are also taken in the logarithmic scale (ln(Old), ln(Hisp) and ln(AA)) and were added to form a multiple regression model, and it was also estimated weekly.

For both regression models, we performed diagnostic tests for the presence of spatial autocorrelation. Although spatial autocorrelation is significant for both the error and spatial lag terms, the error term is stronger in significance in both types of models. Therefore, both the simple and multiple regression models with the spatial error specification were also estimated [21]. Specifically, the spatial error model has the following structure:

MA=Xβ+u,whereu=λWu+ε (1)

In this spatial error model, *MA* is the vector of ma(lct) values, X is the matrix of dependent variables with *β* as the vector of parameter estimates, *u* is the spatial autocorrelated error term and *ε* is the uncorrelated error term. The parameter λ (lambda) is thepatial autocorrelated parameter for the error term. The spatial weights in the matrix *W* are binary with 0 indicating that the pair of units are not neighbors, and 1 otherwise. Neighbors are defined using the queen’s case (sharing an edge or a corner). This spatial error model was estimated for the simple regression and multiple regression cases. It is important to note that variables employed in all these models are in logarithmic scale. Thus, the relationships among cumulative infected cases, population density and sizes of the three population subgroups are nonlinear while the model results are interpreted with the logarithmic scale embedded.

## Results

### Spatiotemporal trajectory

**Fig 1:**
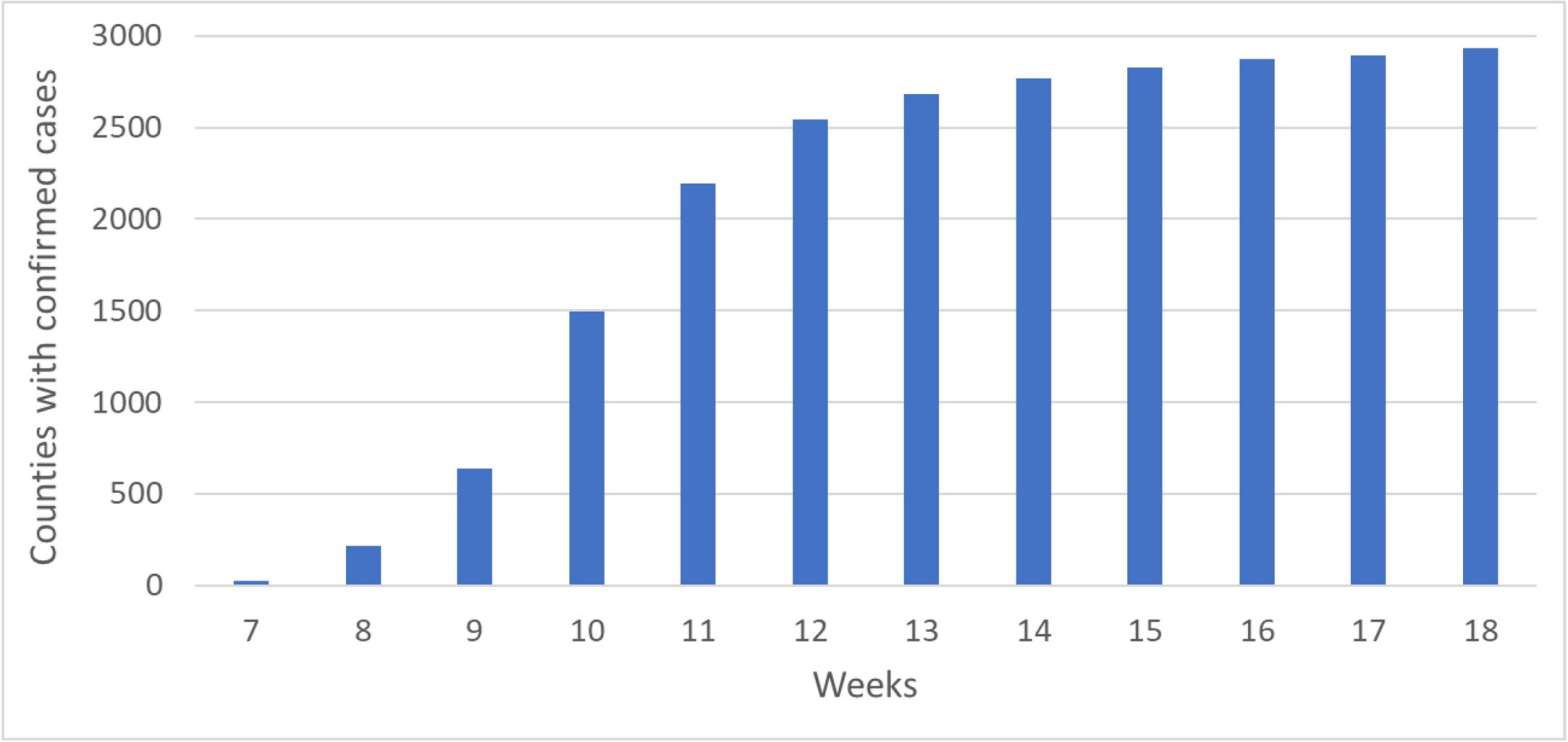
Numbers of U.S. counties with confirmed cases from March 4 (week 7) through May 20 (week 18), 2020.

As mentioned before, due to the limited presence of infection cases in the early phase, our formal analysis starts from week 7 until week 18. The numbers of counties with confirmed cases exploded during the early weeks (Fig 1), increasing from 27 in week 7, to 218 in week 8, 636 in week 9, 1492 in week 10 and 2194 in week 11. Afterward, the numbers of counties with confirmed cases stayed below the 3000 level.

**Fig 2:**
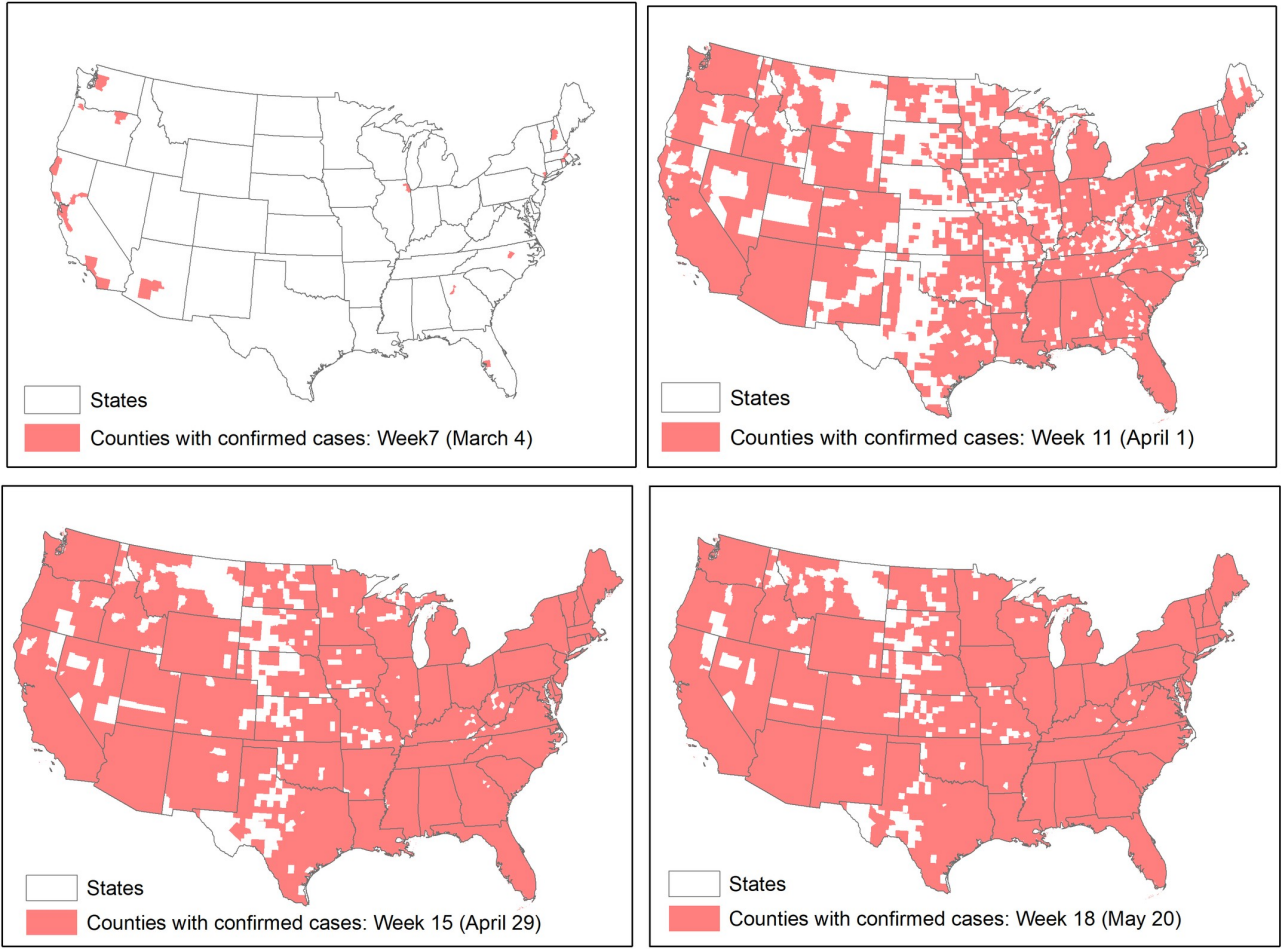
Counties in the continental U.S. with confirmed cases in weeks 7, 11, 15, and 18.

According to our data, the first county with reported cases was King county in Washington state on January 22, 2020. It followed by the counties of Cook (Illinois), Los Angeles, Orange, Santa Clara, San Benito, San Diego, Sacramento and Humboldt (California), Maricopa (Arizona), and Saline (Arkansas). Most of these counties are in large metropolitan areas and have high to moderate population density levels, with the exceptions of Saline, San Benito and Humboldt. The distributions of counties with confirmed cases in weeks 7, 11, 15 and 18 are shown in Fig 2. Starting from a few spots in Week 7, the infection spread to most parts of the continental U.S. in Week 11. From Week 15 to 18, the spatial distributions had been quite stable. Only the central portions from western Texas up to the Northern Plain, and some interior counties could escape from the infection. Most of these counties may be regarded as rural areas.

### Bivariate regression–power of density

**Fig 3:**
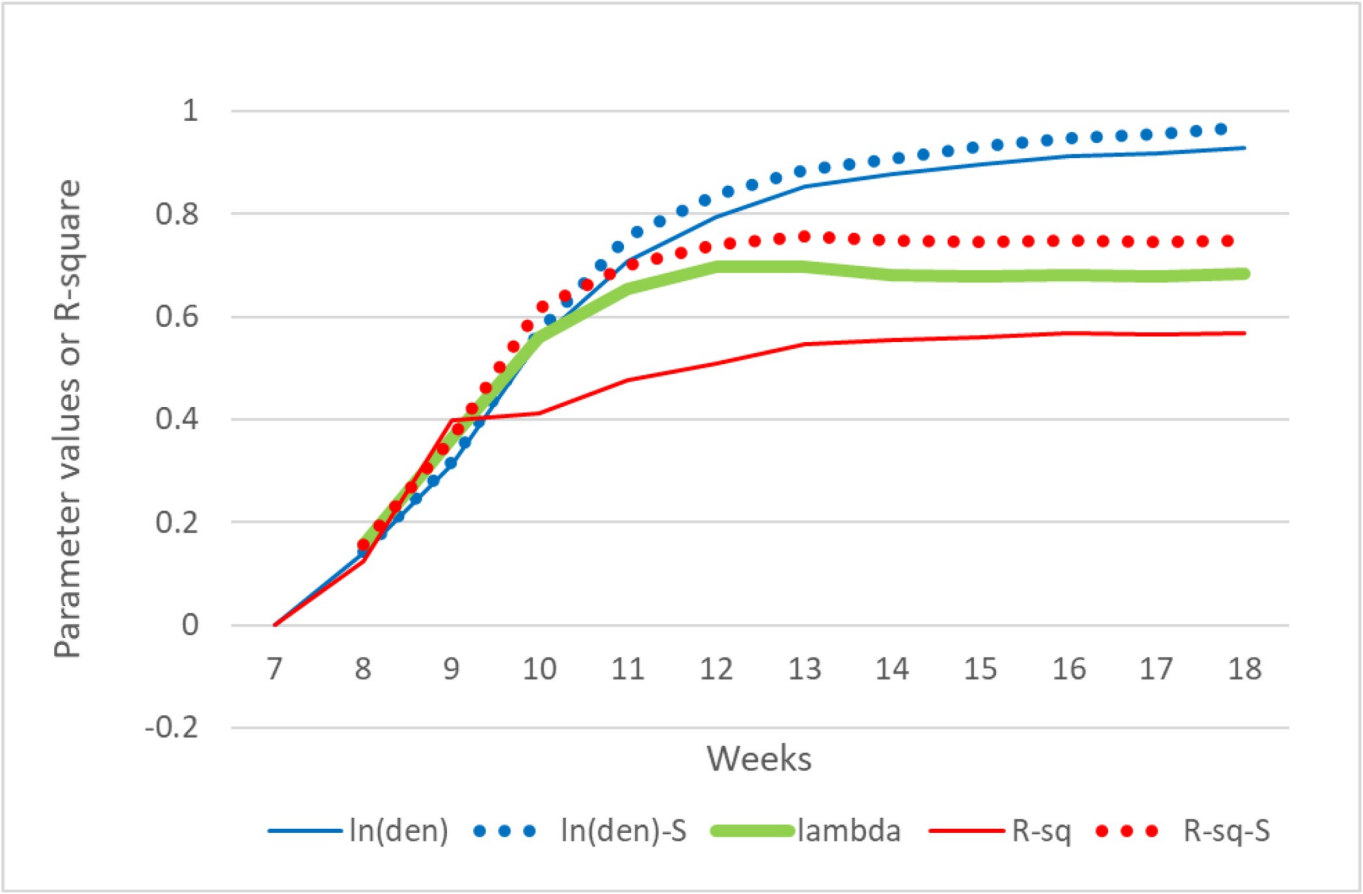
Statistics from the bivariate classical and spatial regression models from March 4 (week 7) through May 20 (week 18), 2020. ln(den) and R-sq are the parameter estimate of ln(den) and R-squared values. The same notations with “-S” appended are their spatial model results. Lambda is the spatial autocorrelation level of the error.

Treating the 7-day moving averages of natural logarithm of confirmed cases (ma(lct)) as the dependent variable and the natural logarithm of density (ln(len)) as the independent variable at the county level, R-squared of the bivariate regression model starts with no correlation in week 7 to 0.57 in week 18. In fact, the R-squared values (R-sq) increase quickly in the first five weeks from 0 to 0.48 in week 11 (Fig 3). Such a trend in R-squared values implies that during the early stage of the outbreak, distributions of confirmed cases across counties were very different from the distribution of population density at the county level. However, as the epidemic progressed, distributions of cases became more similar to the distribution of population density to the extent that about 57% of the variation in ma(lct) can be explained by the variation of ln(den). Fig 3 also shows that over the period, the parameter estimates of ln(den), which are statistically significant except in week 7, also increase, indicating the increasing influence of population density on the numbers of cases.

**Fig 4:**
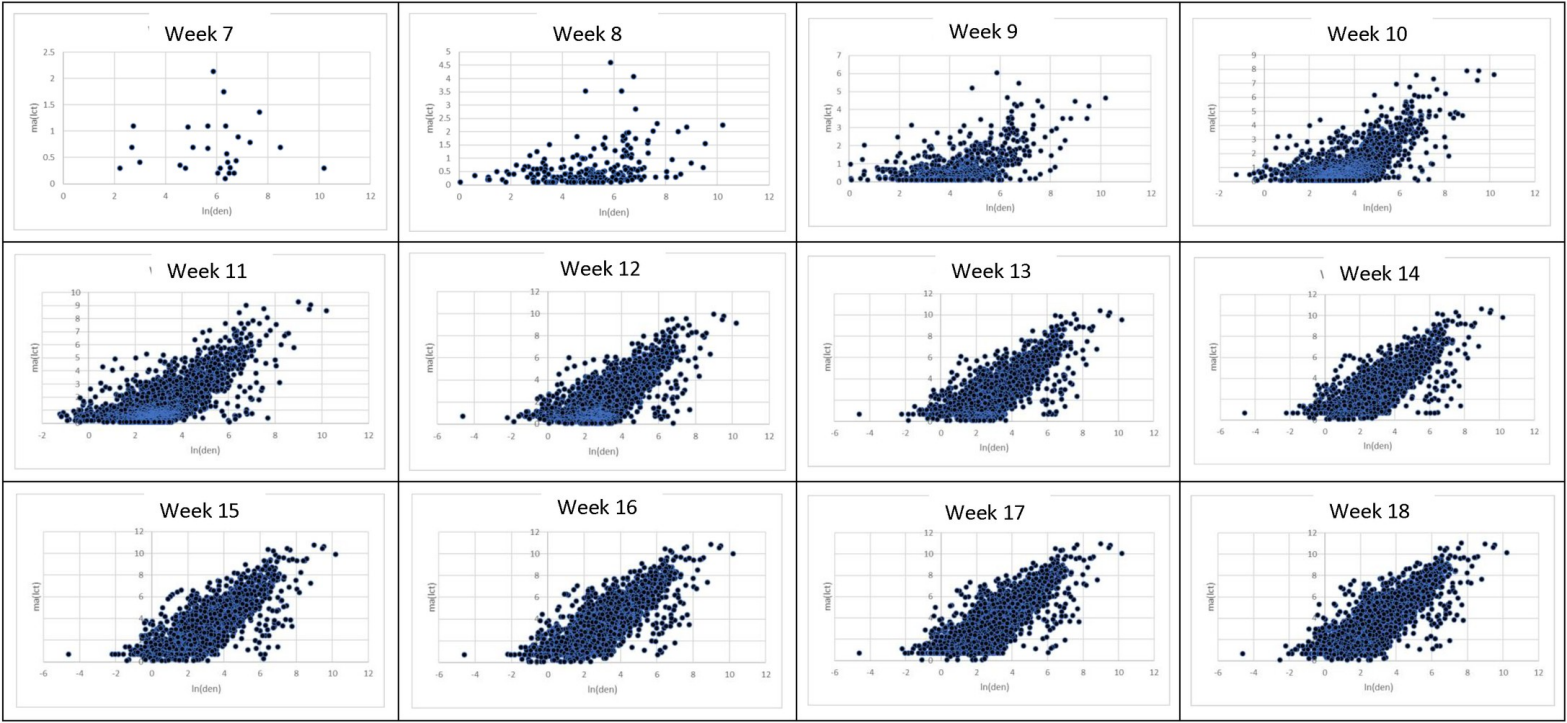
Scatterplots of ln(den) (x-axis) and ma(lct) (y-axis) from March 4 (week 7) through May 20 (week 18), 2020.

Fig 4 provides a visual depiction of the increasing influence of population density on the numbers of confirmed cases as the infection spread across the country from March 4 (week 7) through May 20 (week 18), 2020. In week 7, the relationship between ln(den) and ma(lct) did not exist. In about three weeks (week 10, March 25), their relationship started to emerge. From week 13 (April 15) onward, the relationship solidified. To a large degree, population density constrained the growing numbers of cases across the country.

We also tested if the assumption of regression, particularly the independence of residuals, is violated. Except for week 7, the simple regression models for all weeks have significant spatial dependency. Diagnostic tests show that for most models, the spatial lag of the dependent variable and spatial autocorrelation in the error term are significant, but the autocorrelation in the error term seems to be stronger. Therefore, a spatial error model (Eq 1) was adopted to account for the spatial autocorrelation. Results of the spatial regression models are reported also in Fig 3. By accounting for the spatial spillover effect captured by the spatial autocorrelation parameter lambda, the R-squared values for the spatial error models are as high as 0.76 (in week 13). After the R-squared value reaches the maximum, it leveled off but maintained at the level of around 0.74. The lambda parameter reflecting the spatial autocorrelation of the error terms follows a similar pattern, but peaks in week 12. As Fig 1 indicates, the numbers of affected counties increased quickly before week 12, and the increase slowed down after weeks 12. Fig 2 shows that the distributions of affected counties have not changed much after week 11, the spillover effect captured by lambda leveled off in about week 12.

However, the parameter estimates of ln(den) continued to increase throughout the entire period for both the classical and spatial regression models, even when lambda leveled off in the latter weeks. The continued increase of the ln(den) parameters in both types of models, despite the leveling off of lambda, indicate that the influence of population density continued to increase, even though much less in the latter weeks than in the earlier weeks.

### Multivariate regression–is density sufficient?

**Fig 5:**
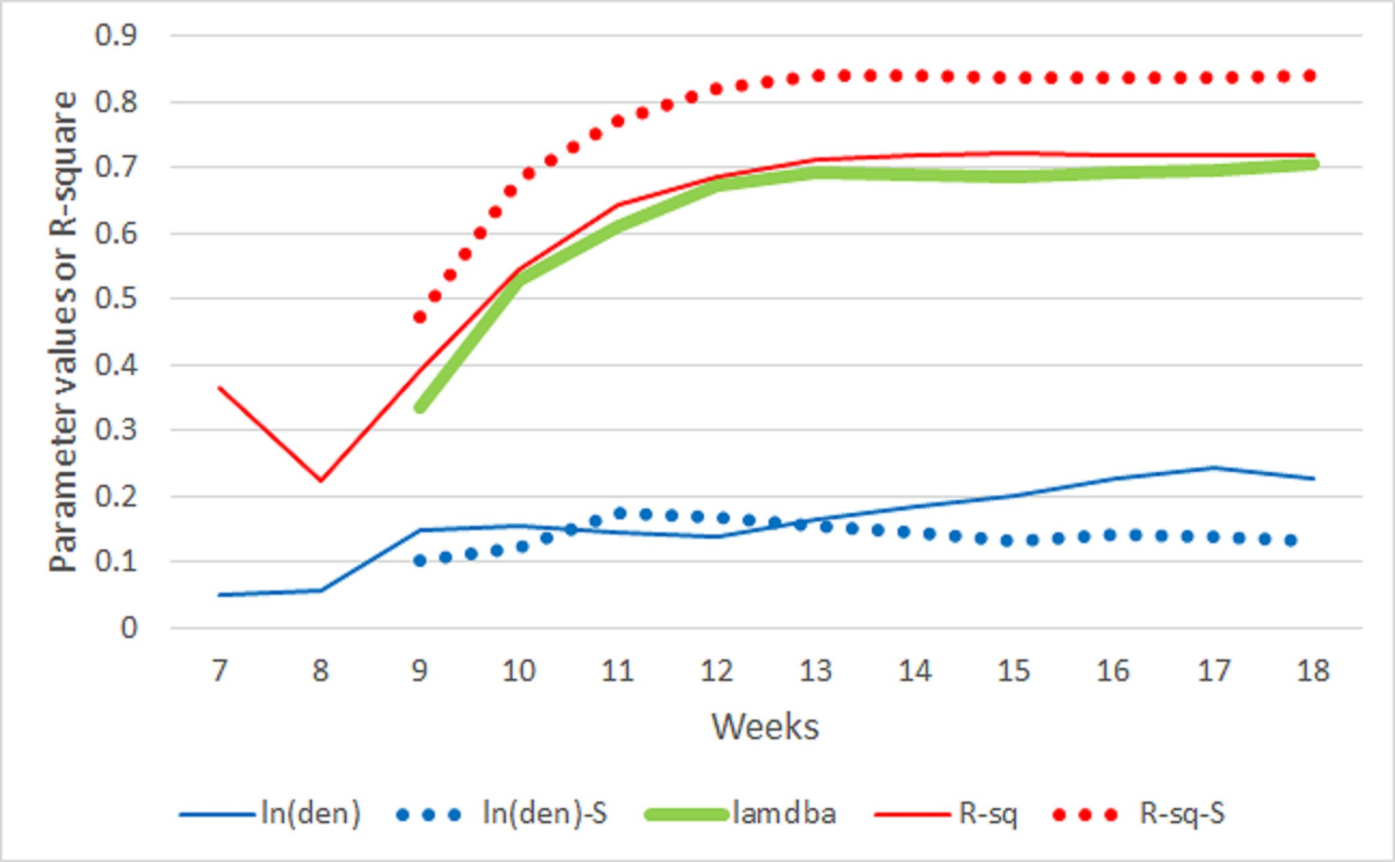
Statistics from multiple classical and spatial regression models from March 4 (week 7) through May 20 (week 18), 2020. ln(den) and R-sq are the parameter estimate of ln(den) and R-squared values. The same notations with “-S” appended are their spatial model results. Lambda is the spatial autocorrelation level of the error term.

**Fig 6:**
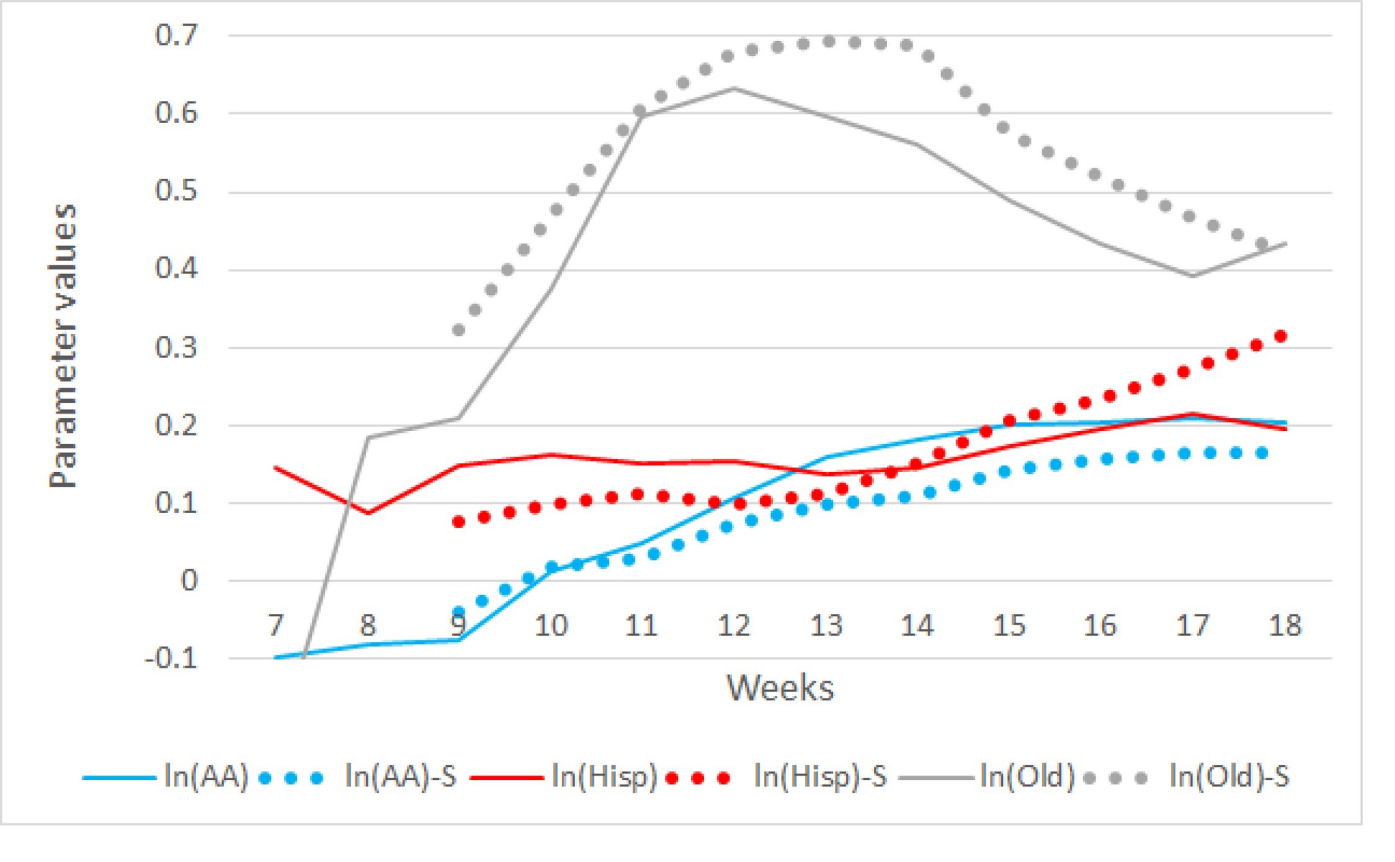
Statistics from multiple classical and spatial regression models from March 4 (week 7) through May 20 (week 18), 2020. ln(AA), ln(Hisp) and ln(Old) are the parameter estimates of the logarithms of the population counts of African American, Hispanic-Latina, and older adults. The same notations with “-S” appended are their spatial model results.

As mentioned before, many reports indicated the disproportional burdens of COVID-19 on several population subgroups: African American, Hispanic-Latina, and older adults. With the logarithms of the sizes of these subgroups, plus the original variable of ln(den), multiple regression models were estimated and results are reported in Figs 5 and 6. Because of the small number of counties with cases in week 7, their statistical estimates are not significant. After week 7, the R-squared values increased over time reaching 0.72 in week 14, and maintained at that level until the end of the study period (week 18) (Fig 5). Comparing to the bivariate aspatial regression models, the R-squared values for the multivariate models are higher by approximately 28%. Different from the bivariate models, in which the parameter estimates of the ln(den) variable increase over time to relatively high levels, from 0.14 in week 8 to 0.92 in week 18, parameter values of ln(den) in the multivariate models do increase, but only slightly from 0.15 in week 9 to 0.24 in week 17, and they are much smaller than those in the bivariate regression models. These differences in the impacts of population density are mainly attributable to the additional variables in the multivariate models accounting for the variation in the dependent variable, ma(lct).

Fig 6 reports the parameter estimates of the three additional variables used in the multivariate models. Except for those models in the earlier weeks (week 7 and week 8), all estimates are statistically significant. Values of these significant estimates varied over time. In week 8, they all started low but with estimates of ln(Old) the largest among the three, and it jumped sharply in subsequent weeks. These results reflect the situations of the U.S. in early weeks very well as some of the earliest outbreaks in the U.S. were originated from older-adult facilities. The increase of ln(Old) estimates slowed down between weeks 11 and 12, and then gradually declined, reflecting that the contributions of cases from older-adult communities had declined over time.

The declining contributions of older adults cases as reflected by the lowering estimates of ln(Old) after week 11 was accompanied by the increasing trends of the ln(Hisp) and ln(AA) estimates, which are both statistical significant. Although the estimates of ln(Hisp) increased slightly over time, bouncing between 0.1 and 0.21, the trajectory of the estimates of ln(AA) has been clearer. It started at slight negatively but consistently moving higher to 0.2 in later weeks. These results indicate that although the influences of the sizes of the two minority groups were different at the start of the epidemic, their influences were about the same levels in later weeks. The size of African American population has become more influential over time while the contributions of Hispanic-Latina population to the morbidity impacts of the epidemic has increased only slightly over the course.

Similar to the bivariate models, we also tested if the multivariate models violate the independence assumption. The diagnostic tests show that all models have a significant spatial dependency in the error term. Thus, the spatial error model (Eq 1) was employed with multiple independent variables. Results of these spatial regression models are also included in Figs 5 and 6. Similar to the bivariate cases, the spatial multivariate models yield higher R-squared values with a maximum of 0.84 in week 18, about 16% higher than the highest aspatial regression R-squared value (Fig 5). The estimates for ln(den) in the spatial models are in general slightly lower than those in the aspatial models, but both are in the range of 0.1 and 0.2. These results may imply that, as compared to the influences of the sizes of population subgroups, the influences of population density levels of a county and its neighbors on the number of infection cases, despite statistically significant, have been modest and relatively stable over time.

Parameter estimates of the three population subgroups in the spatial models follow the general trends of estimates of the three subgroups in the aspatial models (Fig 6). The estimates of ln(Old) in the spatial models are larger than their corresponding estimates in the aspatial models (dotted gray lines are above the corresponding solid gray lines in Fig 6). These results reflect that the concentration of older adults population beyond the county but at the regional level has more influence on the numbers of cases than just the population sizes of older adults in individual counties, indicating the spatial spillover effect related to the older adults concentration.

However, the trajectories of estimates of the other two population subgroups between the spatial and aspatial models are quite complicated. For ln(Hisp), the estimates of the spatial models were smaller than those of the aspatial one in early weeks, but they crossed around week 13 (Fig 6). Since then, the estimates of the spatial models followed an upward trajectory, passing 0.3 in week 18. These trajectories may reflect the changes from the relative local outbreaks (in dispersed counties) to increasing widespread infection at the regional scale in Hispanic-Latina concentration regions such as Arizona, Florida and Texas. On the other hand, estimates of ln(AA) experienced the opposite situation. In early weeks, estimates of the spatial models were slightly higher than those of the aspatial models, but the estimates of aspatial models surpassed those of the spatial around week 10 and they maintained higher than the estimates of spatial models for the remaining weeks with a slight increasing trend. The results may depict the situation that the spread of infection among the African American population had been limited to county scale, not at the regional level across multiple counties.

## Discussion

### Density matters

“Density is destiny” is probably an overstatement in general [22], but our analysis shows that although population density had little to explain the numbers of confirmed cases at the county level during the early stage of the outbreak in the U.S., it became a very competent predictor of the numbers of cumulative cases as the infection spread across the country. With the exceptions of Suffolk county in Massachusetts and Cook county in Illinois with very high population density levels, most counties having the earliest confirmed cases have moderate population density levels. During the intrusion or importation period of the epidemic, earliest cases were likely found in areas with major transportation hubs and their surrounding areas where contracted travelers or the disease vectors resided. These transportation centers and nearby areas do not necessarily have the highest population density levels. As infection cases started in these locations, these counties might have larger numbers of cumulative cases than those counties with later onsets of the epidemic. We have evaluated the influence of early onsets in affecting the number of cumulative cases found in counties throughout the study period (18 weeks) and found that initial infections only influenced the numbers of cumulative cases in early weeks, but the influence dissipated in latter weeks. In addition, the influence of initial cases does not diminish the impacts of population density, spatial autocorrelation (regional effect) and the presence of several population subgroups we studied at the county level. Detailed analysis is reported in S1 Appendix.

As the infectious disease started spreading, places with more people are more likely to have larger numbers of cases. Therefore, population density may not be a significant factor at the early stage of outbreaks, but it is influential in the later stages. The analysis presented here provides strong evidence to substantiate this claim. In addition, our results strongly suggested that besides considering population density at all stages of the pandemic development, local factors such as the presence of certain facilities in the local communities may play some roles in the earlier stages of the pandemic.

Our analysis results also have significant implications on studying and combating other epidemics in general. As shown in the current study, considering only population density can provide a high explanatory power in the variation of cumulative cases for COVID-19. Therefore, it is likely that population density can also be a competent explanatory variable for other airborne infectious diseases. Combating future epidemics caused by airborne infectious diseases similar to COVID-19 should focus on high-density areas.

As mentioned in the introduction section, older adults and racial-ethnic minority groups experienced disproportional burdens. If these demographic and racial-ethnic disparities in morbidity burdens were ignored, the simple bivariate spatial and aspatial models with ln(den) as the only explanatory variable can explain the variations of cumulative cases across counties quite competently. The multivariate models, which are conceptually more comprehensive, provide evidence that the relative sizes of these population subgroups definitely help estimate the impacts of the epidemic.

### Roles of space and boundaries

However, just considering the population density levels and sizes of population subgroups of individual counties are not sufficient. Population concentration and the spread of diseases are not bounded by administrative or statistical units. Our analysis clearly shows that spatial regression models, both the bivariate and the multivariate models, perform better than the classical or aspatial regression models. Besides being more statistically appropriate to account for spatial dependency, these spatial models conceptually consider the situations in neighboring counties. Methodologically, such consideration would moderate a potential problematic issue in our modeling framework. Conceptually, our models explored the relationship between (the log of) cumulative cases and (log of) population density of counties, hypothesizing that high density is related to more cases. Thus, population density reflects the situation of the population over a region–how closely people are packed together. Operationally, demarcations of U.S. county boundaries are often the results of unique local historical developments with little reference to population distribution. Some counties are fundamentally cities with small area but high population density. For instance, the Commonwealth of Virginia has 38 independent cities, most of which are relatively small in area with relatively high population density, but they have an official status equivalent to the other 95 counties in Virginia. Some of them are surrounded by low density counties in the rural regions while some are surrounded by high density counties. On the other hand, some major inner cities are dissected by several counties, each is constrained in area but are highly populated. Examples of these include Washington, DC., and the four New York counties of New York, Kings, Queens and Bronx. Therefore, these high-density counties are part of the integrated urban structure but are “cookie-cut” to form separated administrative-statistical entities. Their population sizes can be large or small, depending on how large the footprints these counties occupy. Mathematically, for high density counties with relatively small to moderate population sizes, our proposed models may predict cumulative infected cases larger than the population sizes of these counties (although predicting total cases is not the purpose of the current study). These anomalies, therefore, may be regarded as artifacts of county boundary delineation, but not the general outcomes of the analysis.

From another perspective, as population density reflects the situation of the population (such as those in the four New York counties), the models conceptually show that highly density environment is associated with large numbers of cumulative infected cases over a region, not necessarily referring to a specific location (point) within the region or bounded by the county boundaries. As spatial models also consider the situations of neighboring units, these small high-density cities or counties are “moderated” by the neighbors’ situations in the modeling framework. If neighboring counties have high density similar to these small high-density counties, the predicted high cumulative counts from the small high-density counties are indicative of the regional situation. If the neighboring counties have density levels much lower than the small high-density counties, the predicted cumulative cases will be suppressed, reducing the impacts of the high-density counties.

Spatial models are also conceptual appropriate to model spatial distribution of diseases. Although counties with high population density levels are expected to have high numbers of cases, these high-density counties may also affect neighboring counties by spreading cases to them. One of the earliest counties having confirmed cases was Saline county, Arkansas with relatively low population density, but it is the western neighbor of Pulaski county where the capital of Arkansas, Little Rock, is located. Therefore, the spatial models can account for spillover cases in counties where their population density may not be high but the population density levels in neighboring counties may be.

On the other hand, population density levels may vary substantially within a county, particularly those peripheral counties of metropolitan areas in which both urban and rural land uses coexist [23]. The statistical models in the current study cannot offer higher R-squared values partly because the county level data fail to capture the intra-county variations. If relatively reliable COVID-19 statistics are available at the sub-county level across the country, models with higher spatial resolutions would be preferred as the COVID-19 transmission also involves spatial processes at the sub-county geographical scales [24]. While population density is a reasonable predictor of the numbers of cases, most prediction models referred to by CDC do not consider population density explicitly [11]. Most of these models use some estimates for contact parameters. Even when population density is considered, only some general measures such as the state-level density are used. However, as argued above, population density level may vary tremendously even within a county. Using the county level population density fails to account for the local situations, not to mention using the state level population density. On the other hand, population density can be computed for multiple geographical scales, even down to the local community and neighborhood levels such as census block groups and blocks. Thus, population density can support the development of high-spatial-resolution prediction models for disease transmission.

Not only population density level varies at the sub-county level, the concentrations of the three population subgroups considered in this study are often manifested as the sub-county level. Our data are too coarse spatially to capture the high concentrations of these groups and their morbidity burdens at the sub-county level [24]. As the county-level analysis here ascertains their significant and substantial influences, their presences at the sub-county level should be considered in future modeling efforts and investigations.

### Beyond density and population subgroups

Although the regression models including the population subgroups variables have moderately high levels of R-squared, substantial portions of variance are still not accounted for. Moreover, after employing the spatial error specification in the models, the regression residuals still have significant spatial autocorrelation, implying that these models still have some missing variables [25]. Some of these missing variables may reflect particular situations of selected counties. For instance, in certain weeks, some counties with relatively high cumulative incident rates have relatively low population density levels. Detailed examination of these counties found that they belong to one of the following situations: counties with a few cases, but with very small population sizes; counties with large numbers of cases from institutions including elderly communities, correction facilities and industrial facilities; counties with small local communities but with visitors and tourists as vectors spreading to the local populations. Removing these counties from the analyses raised the R-squared levels slightly, supporting the notion that population density imposes robust control over the macro-scale structure of the distributions of cases across counties. From a mitigation perspective, these lower-density counties require different sets of policies to avoid the start of community infection and to stop the spread after the outbreak took place locally.

During the outbreak, a term synonymous with COVID-19 is “social distancing” (again, we prefer “physical distancing” or “physical separation.”). As briefly discussed in the introduction, practice distancing is to reduce contact to stop the chain of spreading the infection, but the ability to distancing may be constrained by objective environmental factors well-captured by population density level (personal environmental factors such as living and work arrangements are beyond the scope of the current study). The research presented here identifies the strong positive association between population density and the number of cases. Logically, higher population density makes distancing more difficult. Therefore, this policy may need to be enforced differentially according to population density levels. For areas with high population density, more stringent policies or implementations of distancing may be considered, but for lower density areas, enforcing distancing may not be necessary. Maps in Fig 2 show that as of May 26, 2020, more than 200 counties in the U.S. still did not have confirmed cases and these counties are low population density counties. In other words, low population density offers a strong protective effect against COVID-19 infection and a one-size-fits-all distancing policy may not be effective.

However, distancing is also behavioral. Although high-density environments make distancing more challenging, smart human behaviors may reduce the detrimental effects of high density in the epidemic setting, but ignorant behaviors may amplify damages. People living in high-density environments such as inner cities and major urban centers need to be constantly reminded what to avoid and why. Another major finding in the current study confirms the significant contributions of older adult, African American and Hispanic-Latina population sizes toward infection cases. The presence of these three population subgroups together with a high-density environment could have a synergetic effect on infection spread. Local communities or geographic regions possessing these characteristics should enforce distancing policies stringently and closely monitor for potential outbreaks.

Density matters, and so are the presences of the more vulnerable population subgroups in influencing the impacts of the epidemic. Results reported here calls for the considerations of these factors in developing prediction and assessment models. In addition, these factors can also facilitate the development of models with higher spatial resolutions to predict local sub-county level situations.

## Supporting information

S1 Appendix (DOCX)
