# Supplementary material for: Spreading of COVID-19: Density matters
Source: PLoS One. 2020 Dec 23;15(12):e0242398. doi: 10.1371/journal.pone.0242398 (PMC7757878; doi:10.1371/journal.pone.0242398)
Supplement: S1 Appendix — (DOCX) [file pone.0242398.s001.docx]

***PLoS One* Supporting Information Appendix S1**

Article title: Spreading of COVID-19: Density matters

Authors: David W.S. Wong ^1^, Yun Li ^1*, 2^

The following Supporting Information is available for this article:

**S1 Fig A. Parameter estimates of ln(nweek) variable in the three-variable and spatial regression models from March 4 (week 7) through May 20 (week 18), 2020**. The “-S” appended notation is for the spatial model results.

**S1 Fig B. Statistics from the three-variable and spatial regression models from March 4 (week 7) through May 20 (week 18), 2020**. ln(den) and R-sq are the parameter estimate of ln(den) and R-squared values. The same notations with “-S” appended are their spatial model results. Lambda is the spatial autocorrelation level of the error.

**S1 Fig C. Parameter estimates of ln(nweek) variable in the full multivariate and spatial regression models from March 4 (week 7) through May 20 (week 18), 2020**. The “-S” appended notation is the spatial model results.

**S1 Fig D. Statistics from multiple classical and spatial regression models with the nweek variable from March 4 (week 7) through May 20 (week 18), 2020.** ln(den) and R-sq are the parameter estimate of ln(den) and R-squared values. The same notations with “-S” appended are their spatial model results. Lambda is the spatial autocorrelation level of the error term.

**S1 Fig E. Statistics from classical and spatial multivariate regression models with the nweek variable from March 4 (week 7) through May 20 (week 18), 2020**. ln(AA), ln(Hisp) and ln(Old) are the parameter estimates of the logarithms of the population counts of African American, Hispanic-Latina, and older adults. The same notations with “-S” appended are their spatial model results.

An alternative model specification was experimented based on the notion that counties with early occurrences of COVID cases may have larger numbers of cumulative cases, and counties with later arrivals of COVID have less cases. To reflect the temporal duration of counties with COVID, a new variable nweek was created. Each county is assigned a value (1-18) to the nweek variable to indicate when the first case occurred. Counties with the earliest cases are assigned 18, and counties that did not have a case by week 18 (the last week in the study period) are assigned 1. This variable is added to all existing models, OLS-classical and spatial regression models.

In the three-variable models (OLS and spatial error models), MA, the vector of ma(lct) values (refer to Eq. 1), is the dependent variable with ln(den) and ln(nweek) as the dependent variables. As expected, the numbers of weeks (nweek) in the epidemic is an effective predictor for the first several weeks of the epidemic due to strong temporal correlation of case numbers. Although the nweek variable continues to be a significant factor in explaining case numbers across counties in later weeks, it become less important as the epidemic progressed (Fig. A). Parameter estimates of the nweek variable keep declining after week 11, reflecting the diminishing influence of this variable in later weeks.


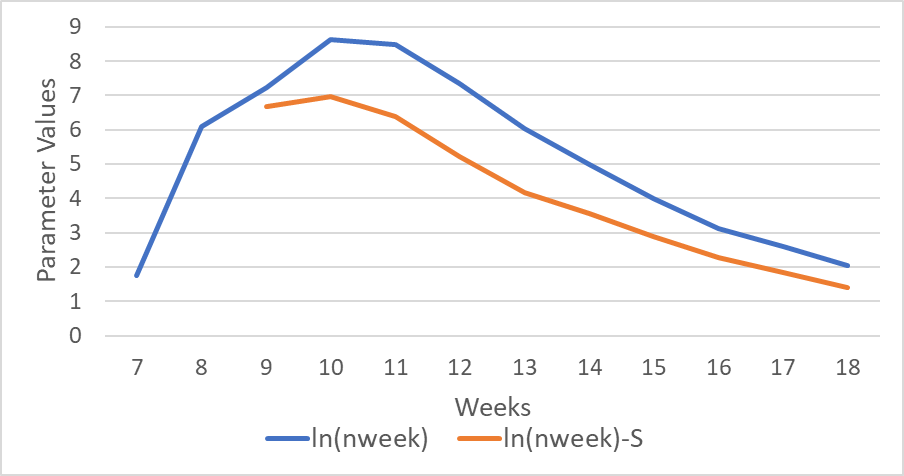


Fig. A. Parameter estimates of ln(nweek) variable in the three-variable and spatial regression models from March 4 (week 7) through May 20 (week 18), 2020. The “-S” appended notation is for the spatial model results.

By including the new variable nweek in the three-variable models, their R-squared values are much higher than the models without the nweek variable in early weeks (weeks 7 to 12), but are just moderately higher for models in latter weeks (Figure B, which can be compared with Figure 3). However, the R-squared values of these models peak in week 13 and decline slightly afterward (Fig. B). These trajectories are different from the increasing trends in the original models without the nweek variable over the entire study period (18). Despite the statistical significance of the nweek variable in both the classical and spatial models, the trajectories of the parameter estimates of ln(den), as shown in Figure B, follow an increasing trend, consistent with the results from the original models that show the increasing importance of population density in affecting the numbers of cumulative cases by counties.


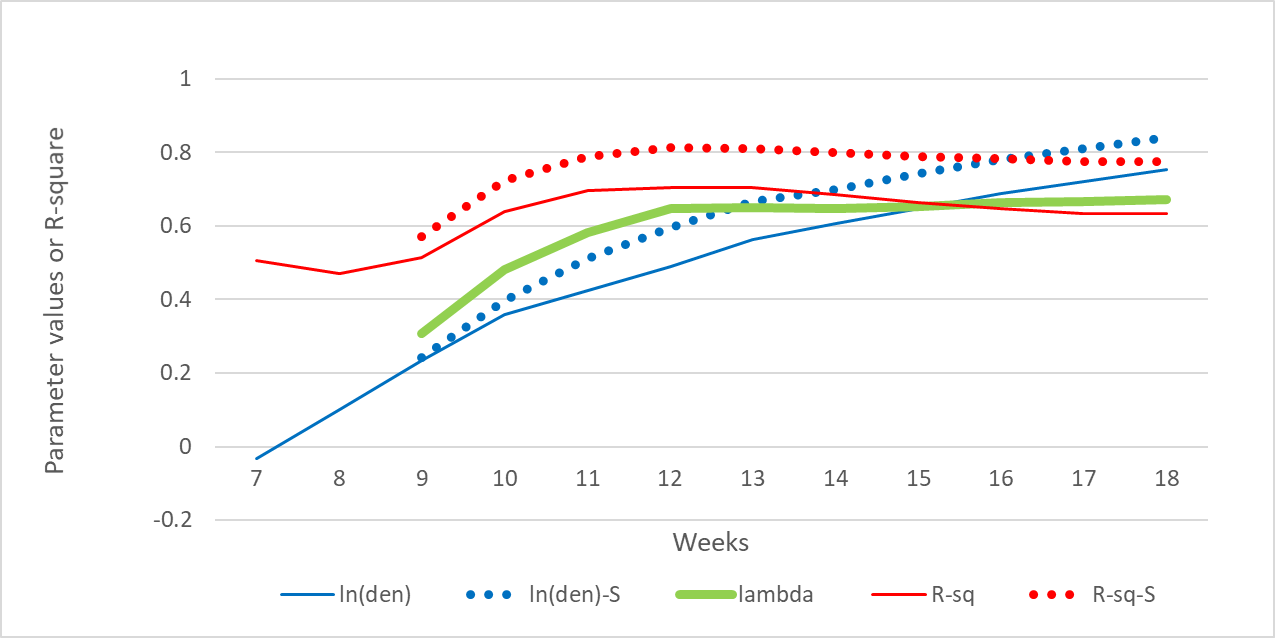


Fig. B. Statistics from the three-variable and spatial regression models from March 4 (week 7) through May 20 (week 18), 2020. ln(den) and R-sq are the parameter estimate of ln(den) and R-squared values. The same notations with “-S” appended are their spatial model results. Lambda is the spatial autocorrelation level of the error.

In the multivariate models that include all population subgroup variables, the new variable nweek becomes less important as the epidemic progressed (Fig. C), exactly the situations found in the simple three-variable models discussed above. The magnitudes of the parameter of the nweek variable keep declining after the week 11, again, reflecting the diminishing power of this variable.


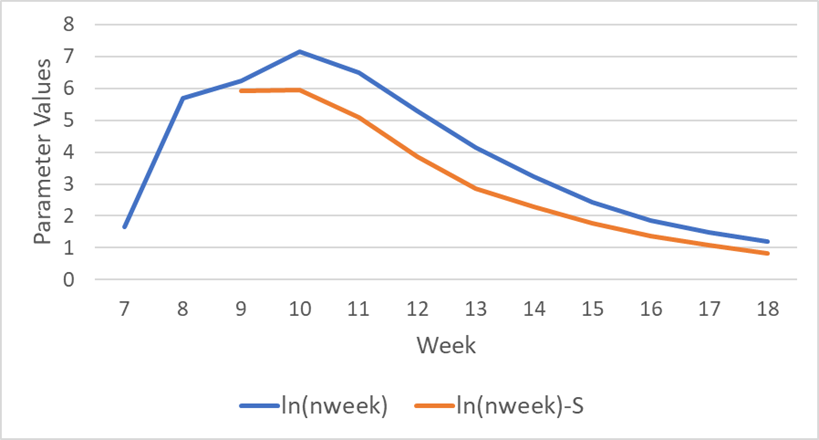


Fig. C. Parameter estimates of ln(nweek) variable in the full multivariate and spatial regression models from March 4 (week 7) through May 20 (week 18), 2020. The “-S” appended notation is the spatial model results.

In the full multivariate models, the R-squared values of the models with and without the variable nweek are very similar. However, the R-squared values of the all models with the nweek variable decline after week 11 or 12 (Fig. D), opposite to the increasing trajectories in R-squared values in models without the nweek variable. In other words, including the nweek variable hampers the performance the models in later weeks. However, the trajectories of all other parameters (Fig. D and E) are similar to the results for models without the nweek variable (Fig. 5 and 6).


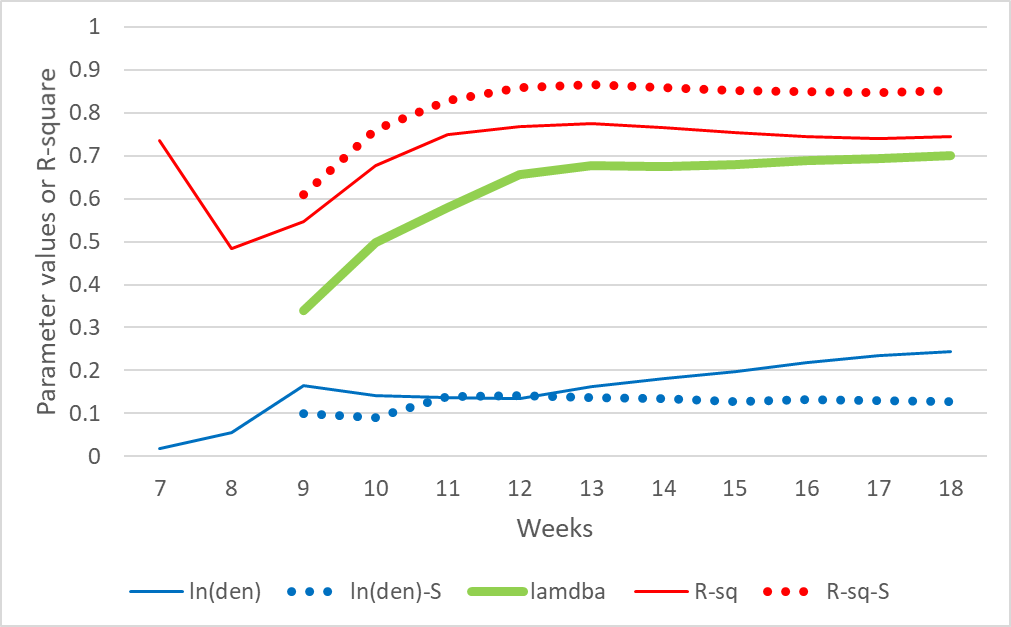


Fig. D. Statistics from multiple classical and spatial regression models with the nweek variable from March 4 (week 7) through May 20 (week 18), 2020. ln(den) and R-sq are the parameter estimate of ln(den) and R-squared values. The same notations with “-S” appended are their spatial model results. Lambda is the spatial autocorrelation level of the error term.


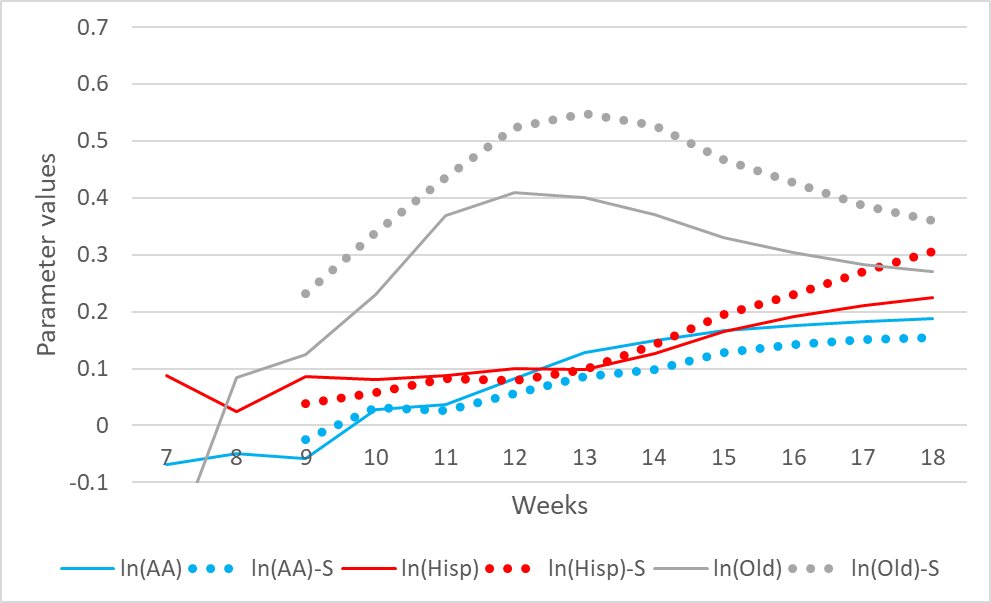


Fig. E. Statistics from classical and spatial multivariate regression models with the nweek variable from March 4 (week 7) through May 20 (week 18), 2020. ln(AA), ln(Hisp) and ln(Old) are the parameter estimates of the logarithms of the population counts of African American, Hispanic-Latina, and older adults. The same notations with “-S” appended are their spatial model results.
